# Idiopathic pulmonary fibrosis beyond the lung: understanding disease mechanisms to improve diagnosis and management

**DOI:** 10.1186/s12931-021-01711-1

**Published:** 2021-04-17

**Authors:** Fabrizio Luppi, Meena Kalluri, Paola Faverio, Michael Kreuter, Giovanni Ferrara

**Affiliations:** 1 Respiratory Unit, University of Milano Bicocca, S. Gerardo Hospital, ASST Monza, Monza, Italy; 2 Centre for Interstitial and Rare Lung Diseases, Pneumology and Respiratory Critical Care Medicine, University of Heidelberg, German Center for Lung Research, ThoraxklinikHeidelberg, Germany; 3 Sensory Motor Adaptive Rehabilitation Technology (SMART) Network, University of Alberta, Edmonton, AB Canada; 4 Division of Pulmonary Medicine, Department of Medicine, University of Alberta, 3-134 Clinical Sciences Building, 11304 83 Ave., Edmonton, AB T6G 2G3 Canada

**Keywords:** Idiopathic pulmonary fibrosis, Comorbidities, Ageing, Senescence, Gastro-oesophageal reflux, Coronary artery disease, Emphysema, Survival

## Abstract

Idiopathic pulmonary fibrosis (IPF) is a chronic and progressive disorder with an estimated median survival time of 3–5 years after diagnosis. This condition occurs primarily in elderly subjects, and epidemiological studies suggest that the main risk factors, ageing and exposure to cigarette smoke, are associated with both pulmonary and extrapulmonary comorbidities (defined as the occurrence of two or more disorders in a single individual). Ageing and senescence, through interactions with environmental factors, may contribute to the pathogenesis of IPF by various mechanisms, causing lung epithelium damage and increasing the resistance of myofibroblasts to apoptosis, eventually resulting in extracellular matrix accumulation and pulmonary fibrosis. As a paradigm, syndromes featuring short telomeres represent archetypal premature ageing syndromes and are often associated with pulmonary fibrosis. The pathophysiological features induced by ageing and senescence in patients with IPF may translate to pulmonary and extrapulmonary features, including emphysema, pulmonary hypertension, lung cancer, coronary artery disease, gastro-oesophageal reflux, diabetes mellitus and many other chronic diseases, which may lead to substantial negative consequences in terms of various outcome parameters in IPF. Therefore, the careful diagnosis and treatment of comorbidities may represent an outstanding chance to improve quality of life and survival, and it is necessary to contemplate all possible management options for IPF, including early identification and treatment of comorbidities.

## Introduction

Idiopathic pulmonary fibrosis (IPF) is a chronic and progressive disorder with an estimated median survival time of 3–5 years after diagnosis. The diagnosis of IPF requires the presence of a typical pattern of usual interstitial pneumonia (UIP) by either high-resolution computed tomography (HRCT) or histology in an appropriate clinical setting and the absence of an identifiable aetiology [1].

Cohort studies suggest that different IPF phenotypes exist [2], with some patients experiencing long periods of stability [3] while others experience exacerbation [4–6] or a rapid progressive decline [7].

Two antifibrotic drugs, pirfenidone and nintedanib, have been shown to slow the decline in lung function in patients with mild to moderate disease [8, 9], but there may be other possible interventions to improve the prognosis of IPF patients [10, 11].

IPF is primarily a disease of elderly people, and epidemiological features suggest that multiple risk factors including ageing and genetic alterations that enhance susceptibility to the disease, as well as environmental factors, especially cigarette smoke exposure, could underlie the pathogenesis of the disease and its comorbidities. Furthermore, although IPF is considered a disease limited to the lung, its risk factors are shared with a number of comorbidities (e.g., cardiovascular and degenerative diseases [12, 13]) that may play an important role in the course of the disease of patients with IPF.

Ageing is defined as a progressive decline in physiological function leading to an increase in the age-specific mortality rate [14]; it is a complex process, involving multiple mechanisms at different levels. The current view suggests that, over time, cells tend to accumulate damage that is intrinsically random in nature and that is normally compensated by genetic mechanisms of maintenance and repair. As cellular defects accumulate, the effects on the body as a whole are eventually associated with age-related frailty, disability and disease [15].

The two main risk factors for IPF, i.e., ageing and cigarette smoke, are associated with common comorbidities, defined as the occurrence of two or more disorders in a single individual [16] (Table 1).

**Table 1:** Frequent comorbidities and medical conditions in IPF patients

Pulmonary	Extrapulmonary
Pulmonary arterial hypertension	Coronary artery disease
Emphysema	Anxiety and depression
Obstructive sleep apnoea	Deconditioning & sarcopenia
Lung cancer	Osteoporosis and bone fractures
Venous thromboembolism	Diabetes mellitus and hypothyroidism
Chronic obstructive pulmonary disease	Gastro-oesophageal reflux

Early evidence suggests that the prevalence and clinical impact of these comorbidities are likely to increase as survival in IPF improves with current and future interventions. However, evidence from pharmaceutical trials is not always fully applicable to the general IPF population because the most influential selection bias in pharmaceutical cohorts is the exclusion of patients with significant comorbidities [17].

In this review, we will highlight the importance of ageing; its mechanisms; and its interaction with other risk factors, such as cigarette smoke, in determining IPF and its comorbidities, highlighting how conditions directly or indirectly related to pulmonary fibrosis may have a similar origin and how their management requires specific diagnostic approaches and combined interventions.

## Molecular basis of IPF: how ageing and cigarette smoke interact

Ageing is a complex process that includes genomic instability, telomere attrition, epigenetic alterations, loss of proteostasis, deregulated nutrient sensing, mitochondrial dysfunction, cellular senescence, stem cell exhaustion, and altered intercellular communication [15]. In particular, cellular senescence is defined as a state of stable growth arrest in combination with distinctive phenotypic changes that include alterations in chromatin and in the secretome [18]. A number of stimuli, including cigarette smoke [19], can mediate senescence via well-described signalling networks that converge on tumour suppressor pathways to induce stable cell cycle arrest [20].

Currently, the most characteristic pathogenetic IPF features are considered the aberrant activation of alveolar epithelial cells, probably determined by environmental factors such as cigarette smoke or air pollution [21], and the accumulation of fibroblasts and myofibroblasts, leading to excessive production of extracellular matrix [22]. In this scenario, ageing is considered a strong risk factor for IPF development, although the mechanisms that link ageing to environmental factors (such as cigarette smoke) in the development of the disease are still largely unclear [23].

A variety of cell types in the lungs of IPF patients undergo ageing and its consequences [24, 25]. Ageing affects both innate and adaptive immunity, impairing cellular defence mechanisms against pathogens and environmental insults such as cigarette smoke [14]. The differences in tissue remodelling in IPF may ultimately be dependent on the specific cell types involved or on their resulting fate (apoptosis versus apoptosis resistance) [14]. Furthermore, the exhaustion of cell populations (such as stem cells and pericytes) in the lung may contribute to the loss of homeostasis between epithelial and mesenchymal cells, leading to fibroblast activation and proliferation [26].

Current evidence suggests that the main hallmarks of ageing occur prematurely in IPF and primarily affect epithelial cells [22]. In fact, alveolar epithelial cell death may be at least partially responsible for abnormal re-epithelialization, epithelial dysfunction, and loss of parenchymal architecture [22]. Furthermore, ageing may have a profound effect in the lung microenvironment through the secretion of a wide variety of mediators that contribute to the development and perpetuation of fibrotic scarring [22]. Alveolar type II cells in IPF lungs also exhibit marked accumulation of dysmorphic and dysfunctional mitochondria and impaired autophagy, which may also contribute to aberrant fibrosis [27]. A decline in mitochondrial function and autophagic activity are common denominators in age-related diseases.

More recently, in addition to alveolar epithelial cells, fibroblasts/myofibroblasts within fibroblastic foci showed markers of cell senescence [25]. Senescent fibroblasts are mostly non-proliferative and show little evidence of apoptosis, which may contribute to their impaired elimination [28].

**Fig. 1:**
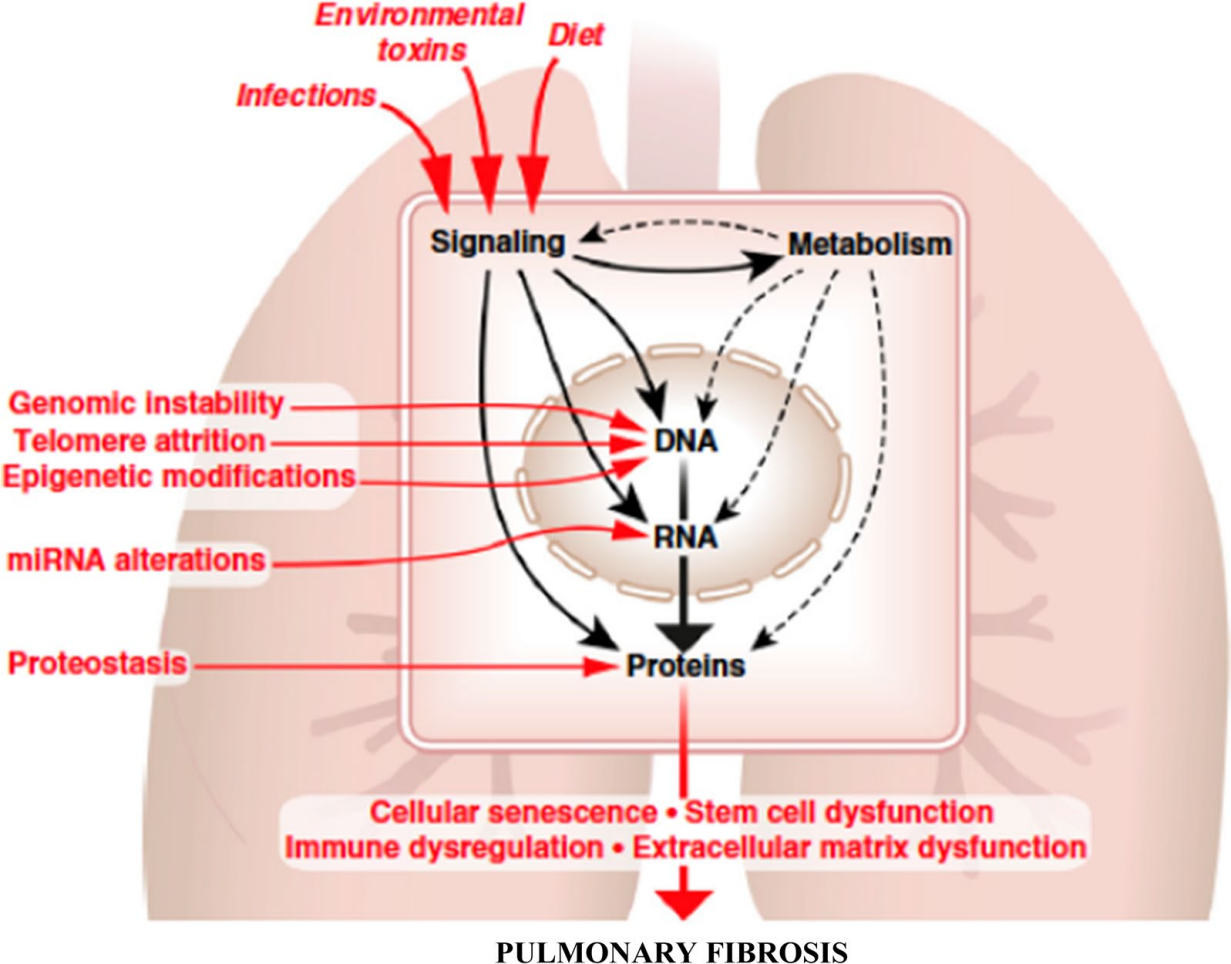
Molecular-level hallmarks of ageing that lead to pulmonary fibrosis ( modified from reference [14])

In conclusion, ageing and senescence, through interaction with environmental factors, may contribute to the pathogenesis of IPF through dual mechanisms, causing the abnormal secretory pattern of the lung epithelium and increasing the resistance to apoptosis in myofibroblasts, resulting in extracellular matrix accumulation and pulmonary fibrosis (Fig. 1).

## Accelerated ageing: the importance of telomeres and other gene mutations and their clinical effects

Telomere length physiologically shortens with age and predicts the onset of replicative senescence. Telomerase is a significant enzyme that maintains chromosome ends [29]. Familial interstitial pneumonia usually occurs at a younger age than non-familial IPF [30]. Furthermore, mutations in the essential genes coding for the enzyme telomerase are the most commonly identified mutations in IPF [31]. Telomerase has two essential components: telomerase reverse transcriptase (hTERT) and hTR, a specialized RNA that comprises a template for telomere repeat addition. At least one of the potential reasons for the difference in age between familial and sporadic pulmonary fibrosis has arisen from the recent description of germline mutations in the genes hTERT and hTR, which are associated with the telomerase complex [32]. Mutations in telomerase and telomere genes distinguish dyskeratosis congenita, a rare syndrome of premature ageing identified a century ago [33]. Approximately one in five patients with dyskeratosis congenita eventually develops pulmonary fibrosis, and telomerase mutations may be found in approximately 15% of patients with familial pulmonary fibrosis [34]. Mutations in a number of telomere-related genes, including TINIF2, nuclear assembly factor 1 (NAF1), dyskerin pseudouridine synthase 1 (DKC1) and regulator of telomere elongation helicase 1 (RTEL1), have been identified and associated with IPF and other interstitial lung diseases (ILDs) [35]. A history of tobacco smoking is present in over two-thirds of affected patients with these mutations [36]. In fact, current and former smokers have shorter telomeres than age-matched controls [37], including in the alveolar epithelium [38], and sex hormones regulate telomerase activity [39], which may contribute to more frequent pulmonary fibrosis in males.

According to the current concept, mutations in telomerase and telomere components predispose adults to a broad spectrum of diseases characterized by pulmonary fibrosis, liver fibrosis and haematological features [40], with age of onset and severity determined by telomere length. Although the exact pathophysiological mechanisms are not known, the loss of telomerase activity may contribute to pulmonary fibrosis through the suppression of fibroblast-to-myofibroblast differentiation [41] and through alveolar epithelial cell senescence limiting alveolar repair [42]. In conclusion, syndromes of short telomeres represent archetypal premature ageing syndromes and are often associated with pulmonary fibrosis.

Among other genes that may have a relationship with cell senescence, the rs35705950 variant in the promoter region of the mucin 5B (*MUC5B*) gene was associated with an approximately sevenfold increased risk for IPF development in a genome-wide linkage study [43]. This MUC5B variant has been validated in various studies and is still considered the most significant genetic risk for IPF [44].

Using genome-wide association technology, three common variants (rs111521887, rs5743894, rs574389) in the Toll-interacting protein (*TOLLIP*) gene were found to be associated with IPF, one of which (rs5743894) was associated with a decreased risk of IPF but increased mortality in those with the disease [45].

A recent genome-wide association study in IPF and fibrotic idiopathic interstitial pneumonia (IIP) by Fingerlin et al. enrolled a total of 2492 patients with fibrotic IIPs (most of them IPF) and compared these with over 6000 control subjects. This study showed seven new loci related to genes controlling host defence, cell–cell adhesion and DNA repair, which contributed to the risk of developing fibrosis [46] and are therefore linked to cell senescence.

## The importance of comorbidities and medical conditions associated with IPF

**Fig. 2:**
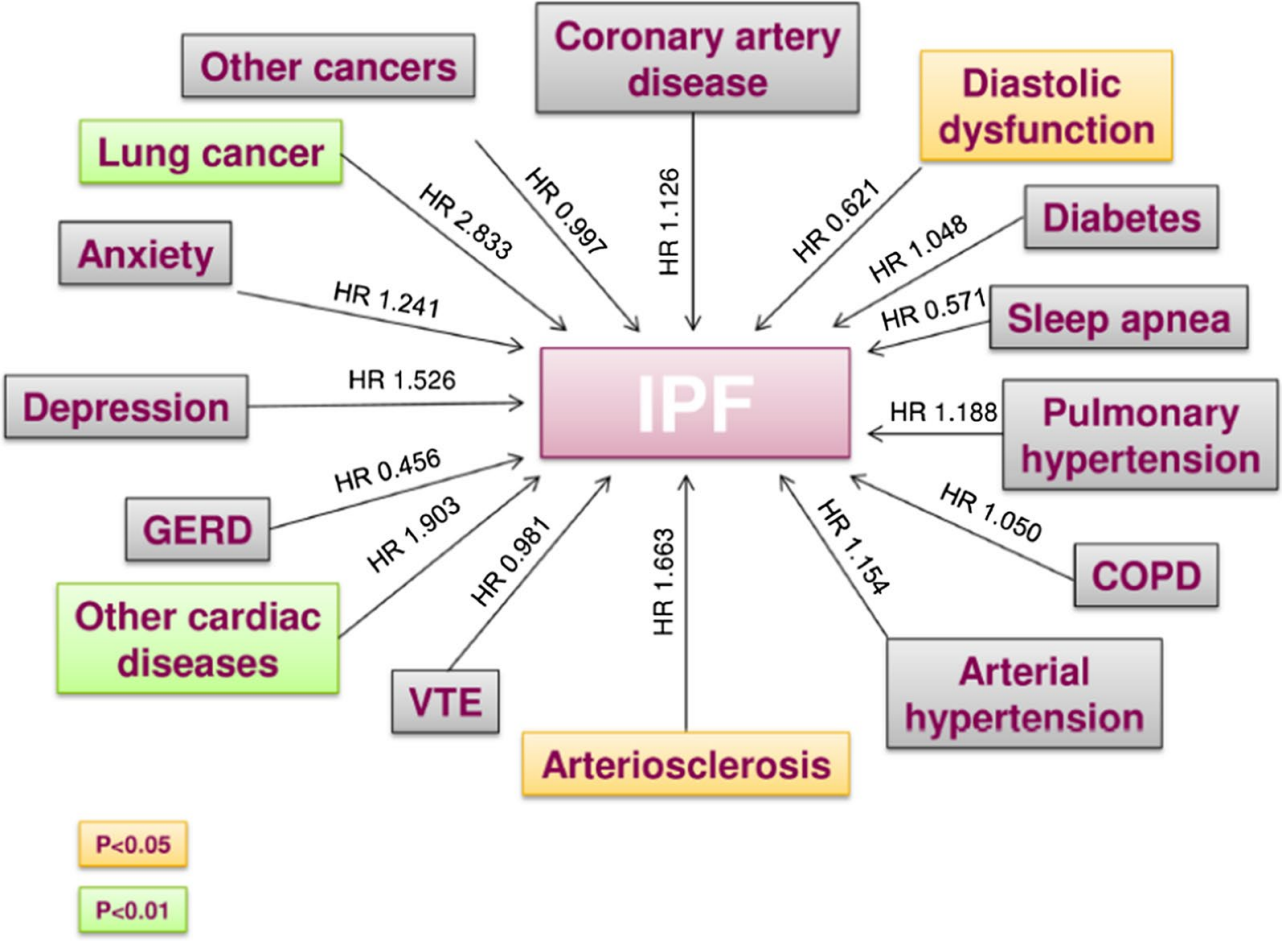
Impact of idiopathic pulmonary fibrosis and comorbidities on mortality (from reference [41])

A recent systematic review confirmed a high prevalence of a variety of comorbidities in IPF patients compared to the general population [47]. Additionally, a recent study reported that approximately 60% of IPF patients showed one to three comorbidities, 30% had four to seven comorbidities and only 10% had no comorbidities, showing that their cumulative incidence is clinically relevant and impacts survival [48] (Fig. 2). Although the prevalence of comorbidities in IPF patients markedly fluctuates with the population studied and the diagnostic criteria utilized, these comorbidities may indeed have a significant impact in IPF, given their additional physiologic and symptomatic effects on the background of underlying pulmonary fibrosis, and they can also potentially characterize other IPF phenotypes.

### Coronary artery disease

Recent studies have shown that coronary artery disease (CAD) is significantly more prevalent in lung transplant candidates with IPF than in those with emphysema (approximately 10% vs 30%, respectively) [49]. In a similar vein, it has been shown that fibrotic lung diseases were associated with an increased prevalence of CAD compared with nonfibrotic diseases after adjustment for usual risk factors [50]. Furthermore, Nathan et al. confirmed the association of IPF with CAD by comparing IPF patients with a control cohort of chronic obstructive pulmonary disease (COPD) subjects, confirming that severe CAD was more common in IPF patients than in COPD patients and was associated with increased mortality [51].

High-resolution CT of the chest is recommended for all patients with (suspected) IPF as a mandatory tool for an IPF diagnosis; it can therefore also become an appropriate tool of screening for CAD in high-risk populations. The detection of moderate-to-severe coronary artery calcifications on high-resolution CT had a high sensitivity and specificity for the diagnosis of CAD [52]. This radiological feature should suggest that clinicians consider a cardiology referral in IPF patients if moderate-to-severe coronary calcifications on HRCT are present.

The high prevalence of this comorbidity in IPF patients is partially explained by a shared genetic predisposition to the development of both IPF and CAD by detection of specific interleukin (IL)-17 immune responses to the alpha 1 chain of collagen type V and by their correlation with human leukocyte antigen (HLA)-D15 alleles [53]. However, smoking history and increased prevalence of diabetes mellitus in these patients, as explained below, may also have a role.

### Other cardiac comorbidities

Low-level evidence suggests that other cardiac comorbidities, including arrhythmias, such as atrial fibrillation, and cardiac or congestive heart failure, may also be frequent in patients with IPF [54]. Data from animal models suggested a potential role of connective tissue growth factor (CTGF) in the remodelling of the heart and of the lung in heart failure [55, 56]. Fibroblasts from IPF patients are known to overexpress CTGF [57]: future studies will elucidate whether this growth factor may influence the occurrence of cardiovascular comorbidities during the natural history of IPF.

### Haematological abnormalities

IPF is associated with a prothrombotic state that may be important in pathogenesis [58]. Consequently, epidemiological studies have also suggested a strong association between IPF and venous thromboembolism (VTE) [59] and consequently with pulmonary embolism [60], which may therefore be considered a comorbidity of IPF. Nevertheless, randomized controlled trials (RCTs) exploring the efficacy of anticoagulants in IPF patients seem to have a detrimental effect on survival [61–63]; therefore, they are not recommended in the treatment of IPF [64].

Mutations in the hTERT and hTR genes are also associated with haematological abnormalities, such as aplastic anaemia, macrocytosis, thrombocytopenia and myelodysplasia/leukaemia [65, 66]. The risk of myelotoxicity/bone marrow failure after lung transplantation is higher in IPF than in other patients, and it should be carefully evaluated in the workup of patients for transplantation [67].

### Osteoporosis and bone fractures

A few studies have explored the relationship between IPF and bone status, showing that reduced bone mineral density has been observed in the thoracic vertebrae of patients who were not receiving corticosteroid treatment [68]. In a recent population-based study, it was shown that approximately one-third of IPF patients have osteoporosis and at least one vertebral fracture. Reduced bone mineral density was associated with significant decreases in FVC and carbon monoxide diffusion capacity (DLCO) [69].

Platelet-derived growth factor (PDGF) subunit B has been linked to both osteoporosis [70] and fibrosing ILD [71], but its role in the pathogenesis of these conditions has not been fully elucidated.

Vitamin D has also been related to bone metabolism. In a recent study, IPF patients exhibited low serum vitamin D concentrations, and such deficiency correlated with all-cause mortality, suggesting its possible role as a prognostic factor and therapeutic target [72].

### Diabetes mellitus

Various studies have shown an increased prevalence of diabetes mellitus in IPF patients [73–75] compared to the general population. In one of these studies, the strongest association was observed with insulin use, and the higher prevalence in IPF patients was sustained even when corticosteroid prescriptions were excluded [74]. The pathogenic mechanisms involved in this association are unclear. High levels of glucose may activate several pathways related to the production of reactive oxidative species and profibrotic cytokines [3] or perhaps, comparable to mucoviscidosis with impaired bacterial clearance in hyperglycaemic states.

Interestingly, abnormal telomere shortening has been found to be associated with type 2 diabetes mellitus, insulin resistance and impaired glucose tolerance [76].

Advanced glycosylation end-products are considered critical for arterial and vascular damage and inflammation in diabetes [77]. RAGE polymorphisms in the receptor for advanced glycosylation end products (RAGE) pathway have been linked to dementia, an increased risk of cancer and an increased risk of cardiovascular disease [78]. The soluble RAGE ligand (sRAGE) counterbalances the activation of RAGE by advanced glycosylation end products.

Furthermore, the nuclear RAGE isoform (nRAGE) seems to be essential to preserve DNA double-strand break repair, and the modulation of this nuclear mediator coupled with an ATM kinase shows the potential to reverse fibrosis in mice [79]. The relationship between the RAGE pathway and the pathogenesis of diabetes and IPF is still unexplored. Nevertheless, the first study exploring the potential role of the RAGE pathway in IPF showed that circulating levels of sRAGE were reduced in IPF patients, especially if they bore the rs2070600 A allele, and it correlated with the severity of the disease [80].

### Hypothyroidism

In a retrospective case–control analysis, Oldham et al. showed that hypothyroidism was common and markedly more prevalent (13% of men and 28% of women affected) among IPF patients than among the general population (1–2% of men and 5–9% of women) [81]. The potential importance of this comorbidity is underscored by the capacity of hypothyroidism to predict mortality in IPF [81].

The relationship between the thyroid and the lung is complex, and its deep mechanisms are still unknown; mutations in thyroid transcription factor NK2 homeobox 1 (NKXS2-1) are linked to brain-thyroid syndrome, affecting neonates and children with histological features of surfactant disruption [82]. Furthermore, the presence of autoimmune hypothyroidism is associated with increased mortality in chronic hypersensitivity pneumonia [83]. Furthermore, anecdotal cases of reversion of pulmonary fibrosis in vivo with initiation of thyroxin replacement have been described, although they remain poorly characterized [84].

### Emphysema

Combined pulmonary fibrosis and emphysema (CPFE) defines the coexistence of upper lobe emphysema and basilar fibrosis. This term was established by Cottin et al. in 2005 [85], although earlier descriptions of this co-existence were reported [86]. Whether CPFE characterizes a unique disease entity or a coincidence of two pulmonary diseases related to cigarette smoking is as yet unclear. The reported prevalence of CPFE is broad, ranging from 8 to 51%, but with a median of about a third of patients with IPF [87] with the possibility to identify a specific phenotype of IPF patient in which CPFE may occur, usually an older male and invariably a current or former smoker, often with marked exertional desaturation [87]. The diagnosis of this condition was established by high-resolution CT of the chest. Patients often have marked exertional desaturation, and pulmonary function tests frequently reveal preserved lung volumes with a severely reduced carbon monoxide diffusion capacity [88]. The combination of opposing obstructive and restrictive volumes determines a preserved lung volume with a severe reduction in diffusing capacity. Pulmonary hypertension (PH) is another common comorbidity [85, 89] and heralds a negative prognosis [90]. However, CPFE is also associated with an increased risk for other comorbidities, including lung cancer. In fact, the prevalence of lung cancer is higher in these patients than in those with emphysema or IPF alone [87]. Survival is significantly worse in patients with CPFE and lung cancer than in those with emphysema and lung cancer alone, suggesting that the “triple hit” effects of smoking, emphysema, and pulmonary fibrosis may be detrimental to CPFE patient prognosis [91]. Another clinically relevant comorbidity of CPFE is the occurrence of acute exacerbations, which are similar to those occurring in IPF patients. Several risk factors may be responsible for the occurrence of acute exacerbations in CPFE patients, including lung resection surgery [87], together with radiation and chemotherapy in patients with concomitant lung cancer.

Interestingly, telomere length and mutations in the TERT/TERC system have been linked to both emphysema susceptibility [92] and to CPFE [93, 94], supporting the concept that both manifestations are part of an ageing process of the lung, determined by genetics and environmental factors.

Because no clinical trial specifically targeting CPFE patients has been performed, treatment recommendations in these patients are based on expert opinion. Smoking cessation, vaccinations, supplemental oxygen, and pulmonary rehabilitation should be prescribed when appropriate. Treatment of CPFE patients with inhaled long-acting anti-cholinergic drugs, inhaled long-acting beta agonists and/or inhaled corticosteroids is of unclear benefit [95]. Patients without contraindications should be referred for lung transplantation assessment when appropriate.

### Anxiety and depression

Since IPF is a chronic and progressive condition with a significant impact on morbidity and mortality, it is frequently associated with anxiety and depression. Specifically, worsening dyspnoea, declining pulmonary function, and hypoxemia are characteristic features of IPF progression, and they have been shown to correlate with depression and anxiety [96], which, together with cough and dyspnoea, are major determinants of health-related quality of life [96] and common comorbidities among IPF patients. The prevalence of anxiety and depression is high (31% and 23%, respectively) [96]; therefore, all patients diagnosed with and followed up for IPF should be screened for these disorders [87]. As a consequence of the underestimation and underdiagnosis, pharmacologic therapy for anxiety and depression was prescribed in only a quarter of cases, suggesting that a large proportion are untreated, despite a high prevalence [97]. Cognitive behavioural therapy and antidepressant medications can be recommended in IPF patients to improve quality of life, although the effectiveness of these treatments has not been validated in IPF patients [87].

Pulmonary rehabilitation has been shown to result in sustained improvements in depressive symptoms and should be recommended to all patients with IPF who have depression and functional impairment [98].

In advanced fibrotic lung disease, a palliative care intervention showed a positive effect on both anxiety and depression, confirming the importance of best supportive care in these patients [11].

### Pulmonary hypertension (PH)

Pulmonary hypertension is defined as a resting mean pulmonary artery pressure (mPAP) greater than or equal to 20 mmHg as assessed by right heart catheterization [99]. A normal mPAP (± standard deviation) is equal to 14 ± 3 mm Hg. PH due to IPF is a frequent comorbidity of the disease, particularly at an advanced stage and is believed to be a marker of poor prognosis [100, 101].

However, a study performed on more than 6500 patients indicated that even mild pulmonary hypertension in patients with IPF increases the risk of death [102]. Some studies also suggest that precapillary PH in IPF may range from approximately 8% to 15% of patients upon initial workup [103, 104], a percentage that becomes higher in IPF patients with advanced and end-stage disease, when PH becomes a common feature (> 50–60%) of the disease [105–107].

The frequency of PH further increases with some comorbidities, such as obstructive sleep apnoea, thromboembolism or cardiac diastolic dysfunction [87].

Generally, the degree of PH in IPF patients is mild to moderate. However, some patients (approximately 10%) show disproportionate pulmonary arterial pressure compared to the severity of lung disease as measured by lung function tests [108] or HRCT fibrosis score [109]. In the past, the discrepancy between the degree of PH and the severity of the lung disease was called “out-of-proportion” PH. Currently, this term has been abandoned since guidelines introduced the concept of severe pulmonary hypertension for patients with mPAP ≥ 35 mm Hg, or mPAP ≥ 25 mm Hg in the presence of a low cardiac output (cardiac index < 2.5 L/min) [110].

Symptoms of PH in patients with IPF are non-specific, and the presence of PH can be easily missed or the diagnosis delayed. In fact, the occurrence of PH in IPF may be characterized by increased dyspnoea, reduction of gas exchange at rest, markedly low DLCO, rapid desaturation upon exercise, high brain natriuretic peptide (BNP) levels, right heart dilation on chest radiography, impaired quality of life, lower exercise tolerance, and greater supplemental oxygen requirements [107]. Rapid progression of PH was observed in late-stage IPF patients [106].

Although right heart catheterization (RHC) is considered the gold standard for PH diagnosis, transthoracic echocardiogram is usually utilized as the initial screening tool for pulmonary hypertension, although its accuracy in chronic lung disease, including IPF, is still a matter of debate [111].

Supplemental oxygen is indicated for the prevention and therapy of PH due to hypoxia, although no data are available supporting the beneficial effect of oxygen on survival in this group of patients.

There are no approved targeted therapies for PH in IPF [112], and recently, a number of negative clinical trials using vasodilator therapies have been performed. Specifically, the use of vasodilator therapies for IPF with or without PH failed to demonstrate efficacy in slowing IPF progression [113–115] and did not modify cardiovascular haemodynamics in those with concomitant PH [116, 117]. A trial using ambrisentan in IPF patients with right heart catheterization-proven PH was stopped after another trial of ambrisentan showed no benefit in the subgroup of IPF patients with known PH [114]. A further trial evaluating riociguat, a soluble guanylate cyclase stimulator, was also stopped after an interim analysis showing that patients in the intervention arm had an increased risk of death and other serious adverse events compared to the controlled arm [118].

Sildenafil, a phosphodiesterase-5 inhibitor, was studied in patients with advanced IPF. In a large double-blind, placebo-controlled trial called “STEP-IPF”, IPF patients were randomized to receive oral sildenafil or placebo. The primary outcome measure was the presence of at least a 20% improvement on the 6-min walking test (6MWT) at 12 weeks: it did not meet statistical significance, as only 10% in the sildenafil arm versus 7% in the placebo arm showed improvement (P = 0.39). However, small but clinically significant differences in the secondary outcomes of arterial oxygenation, DLCO, degree of dyspnoea and quality of life in patients receiving sildenafil were observed [119]. A post hoc subgroup analysis of patients with evidence of PH showed that sildenafil therapy improved walking distance as well [120]. Furthermore, the results of a recent network meta-analysis highlighted potentially important differences in mortality and serious adverse events between different treatment interventions for IPF. These findings suggest a possible mortality advantage of nintedanib, pirfenidone, and sildenafil compared to other treatments [121]. Based on all these data, the most recent evidence-based guidelines conditionally recommend against the routine use of sildenafil in patients with IPF [1], and clinical trials investigating sildenafil in combination with anti-fibrotic therapy—both pirfenidone and nintedanib—for patients with IPF-associated PH have been published [122–124].

A recent phase 2b/3 trial with pulsed inhaled nitric oxide in patients with fibrotic ILDs complicated by PH showed preliminary promising results on a beneficial impact of this treatment on physical activity with a good overall safety profile and tolerability [120].

### Gastro-oesophageal reflux

Gastro-oesophageal reflux (GER) is the reflux of gastric contents into the oesophagus, with some degree of GER occurring physiologically. GER can be acidic or non-acidic with oesophageal pH values below or above 4.0, respectively. Gastro-oesophageal reflux disease (GERD) is a condition that develops when the reflux of stomach contents causes symptoms or comorbidities [125].

It is important to note that GERD is not synonymous with GER or simple heartburn and that GERD syndrome, by definition, involves adverse downstream effects from the reflux [65].

GERD is commonly observed in patients with IPF [126, 127] and more frequent compared with the general population [128] or patients with other chronic lung diseases, with a prevalence ranging from 70 to 90%.

Recently, Ghebre and Raghu [129] hypothesized 2 different mechanisms in establishing a cause-effect relationship between GERD and IPF: (1) chronic micro-aspiration causes recurrent injury to the bronchiolar and alveolar epithelium, and therefore micro-aspiration drives fibrogenesis in susceptible individuals, leading to IPF; (2) decreased lung compliance of the fibrotic lung causes an increase in intrathoracic pressure that leads to a dysfunctional lower oesophageal sphincter, GERD, and micro-aspiration that perpetuate and/or accelerate the IPF disease process.

The typical GERD symptoms, including heartburn, are present in only 25–65% of IPF patients with a confirmed GERD diagnosis; therefore, the absence of symptoms does not preclude a diagnosis of GERD in IPF patients [130].

Although 24-h pH monitoring and oesophageal manometry can be useful in identifying GERD, the best diagnostic flowchart to diagnose GERD in IPF patients is undefined and should therefore be personalized. Mainly for this reason, GERD therapy is often started on the basis of GER symptoms [87].

GERD also seems to be a risk factor for acute exacerbation of IPF (AE-IPF): micro-aspiration is in fact believed to be one of the mechanisms triggering AE-IPF, as demonstrated by Lee et al., who showed a high level of pepsin in bronchoalveolar lavage fluid of AE-IPF patients. The detection of pepsin in bronchoalveolar lavage fluid of these patients was proposed as a biomarker of micro-aspiration and correlated with AE-IPF even if its increased level was not predictive of mortality [131].

The benefit of antacid medication on IPF progression is still debated. Case series and uncontrolled trials have shown beneficial effects of the treatment of GERD in IPF, including lung function stabilization in IPF patients affected by GER and treated with antacid therapy [132]. Furthermore, in a prespecified analysis of the IPFnet trials, Lee et al. showed a slower decline in lung function in IPF patients in which GER was diagnosed and subsequently treated with antacid therapy (mainly proton-pump inhibitors, or PPIs) [133]. The updated 2015 evidence-based IPF guidelines stated that the low quality of evidence led experts to make a conditional recommendation for the use of antacid treatment for patients with IPF [64]. In a recent systematic review and meta-analysis, including 8 observational studies, Fidler et al. showed that pharmacologic treatment of GER was associated with a significant reduction in IPF-related mortality compared with no GER treatment but not with all-cause mortality. However, the authors stressed the low quality of evidence for these outcomes and the consequent need for randomized trials exploring the effect of antacid therapy in IPF patients [134].

In contrast, a recent pooled, post hoc analysis including patients with IPF from the placebo groups of three trials showing the efficacy and safety of pirfenidone (the CAPACITY 004, CAPACITY 006, and ASCEND trials) could not replicate the effects of PPIs and reported a potential association with an increased risk of infection in those with advanced disease [135]. In a similar vein, post hoc analyses obtained from the INPULSIS trials, showing the safety and efficacy of nintedanib in IPF patients, also suggest that patients treated with PPIs at baseline may actually do worse [63]. The above post hoc analyses leave uncertainty on the possible treatment benefits of antacid treatment for patients with IPF, and Spanish [136] and German [137] guidelines for IPF do not recommend antacid treatment of IPF patients for their primary disease underlying the urgent need for a well-defined, randomized, controlled clinical study to prospectively evaluate safety and therapeutic efficacy of PPIs/antacids and anti-reflux surgery for IPF.

While anti-acid treatments target the acidic component alone, surgical interventions include repair of hiatal hernia and Nissen fundoplication to treat GERD by suppressing the anatomical predisposition to reflux [138].

This strategy acts by suppressing the reflux of both acidic and nonacidic gastric material from the stomach, and thus, the reduction risks of aspiration are clearly reduced [139]. Raghu et al. showed a possible trend towards stabilization in forced vital capacity (FVC) pre- and post-laparoscopic anti-reflux surgery (LARS) over 1 year, which warranted prospective studies [140]. In a first prospective and randomized phase 2 clinical trial, the Weighing Risks and Benefits of Laparoscopic Anti-Reflux Surgery in Patients with Idiopathic Pulmonary Fibrosis trial, studying the safety and efficacy of LARS in patients with IPF with GERD, Raghu et al. showed that LARS was safe and well tolerated. Disease progression was not reduced significantly over time despite the evidence of some clinically meaningful trends. Respiratory-related hospitalization and death were less common in the surgical group, without statistical significance [141].

### Obstructive sleep apnoea

Various studies have shown that obstructive sleep apnoea (OSA), defined by an apnoea-hypopnoea index > 15 events/hour, is frequent in IPF patients [142–144], with up to approximately 70% of these patients having moderate to severe OSA.

Several sleep alterations have been detected in IPF patients, including changes in sleep architecture, such as abnormal sleep stage distribution, multiple awakenings, decreased percentage of total sleep time, low sleep efficiency, and increased wake time after sleep onset, in breathing pattern, such as increased respiratory frequency during sleep and rapid and shallow breathing, especially during rapid eye movement (REM) sleep, and in nocturnal oxygenation parameters, such as oxygen desaturation (during both REM and non-REM sleep) and desaturation due to respiratory events (apnoeas and hypopneas) [145].

The variety of obstructive sleep-disordered breathing in IPF patients comprises a spectrum of respiratory events during sleep ranging from simple snoring to complete cessation of airflow (apnoea) [145]. Considering the high prevalence of sleep-disordered breathing, nocturnal polysomnography should be performed at the time of IPF diagnosis to detect occult OSA. In contrast, a retrospective study performed in IPF patients showed that less than 3% of patients underwent a sleep study [142]. The importance of an early OSA diagnosis in IPF patients is mainly related to the evidence suggesting that untreated OSA can result in nocturnal hypoxemia, which was observed to predict worse survival [146].

The proposed connection between IPF and OSA is probably based on tracheal traction theory. In fact, decreased lung volumes, observed in restrictive pulmonary diseases, can reduce upper airway stability and increase resistance because of decreased traction on the upper airway, therefore facilitating upper airway collapse [145].

Patients with IPF with OSA should be treated with continuous positive airway pressure (CPAP), as are all other patients with OSA, improving quality of life and decreasing mortality in this population [147].

However, a high rate of continuous positive airway pressure (CPAP) nonacceptance or poor compliance, mainly because of claustrophobia, irritating cough during sleep, insomnia, and depression, has been reported in IPF, suggesting the need for follow-up by a well-organized sleep centre [148].

Supplemental oxygen is generally prescribed to correct nocturnal hypoxaemia. However, no studies have demonstrated an effect on long-term survival or on the development of pulmonary hypertension [149].

### Lung cancer

The risk of developing lung cancer (LC) is markedly higher in patients with IPF than in a control population [150]. This increased risk persists even with adjustment for age, sex, and smoking history, suggesting that the lungs of patients with IPF are predisposed to develop malignancies. Interestingly, the cumulative incidence of lung cancer increases markedly over time, from approximately 3.3% at 1 year after IPF diagnosis to 15.4% at 5 years and 54.7% at 10 years. In this study, age at initial IPF diagnosis was a significant independent factor predicting the development of lung cancer [151]. The mechanisms behind the development of lung cancer in IPF have not yet been fully explored, but recent evidence seems to suggest that a common genetic predisposition may exist; mutations in the surfactant protein A1 (SFTPA1) gene were found in patients with IPF and lung adenocarcinoma, and an increased risk of developing interstitial lung diseases or other forms of cancer was found in the members of their families [152]. Interestingly, the RAGE polymorphism rs2070600 of allele A, found in severe IPF [80], may be linked to a higher risk of lung cancer [153].

In IPF, lung cancer often presents with nonspecific symptoms, including haemoptysis, weight loss, and other constitutional symptoms; when detected in IPF patients, they should therefore prompt further evaluations [154]. Additionally, radiological diagnosis may be difficult in these patients given the fibrotic changes in the lungs, as it typically appears as nodular lesions with irregular or spiculated margins in peripheral lung areas that are complex to distinguish apart from fibrotic lesions [155, 156]. In a population of Italian IPF patients, the most frequent histologic subtypes were peripheral squamous cell carcinoma and adenocarcinoma [157].

Although the studies that reported mortality and survival among IPF patients with lung cancer were limited by small sample sizes, survival was decreased in those with concurrent LC compared to patients with either idiopathic interstitial pneumonia [158] or IPF alone [157].

IPF patients with lung cancer are generally not good candidates for standard therapy, particularly because of the decreased tolerance of these patients to cancer therapies. Management of lung cancer in IPF should therefore be approached on a single-patient basis, balancing the likelihood of treatment against the potential for therapy comorbidities and considering the frequent poor prognosis of IPF [154]. Patients with pulmonary fibrosis are at high risk of mortality following thoracic surgery, including lung cancer resection [159]. This increased risk is often secondary AE-IPF, which occurs in approximately 10% of IPF patients following thoracic surgery, with a high subsequent short-term mortality (approximately 50%) [6]. The exact mechanism by which surgical procedures may trigger an AE is presently unknown.

AE-IPF was also observed in patients treated with chemotherapy for lung cancer or following radiotherapy [160, 161]. A recent European survey on the management of lung cancer in patients with IPF reported stereotactic radiotherapy as the preferred therapeutic approach for 54% of respondents in those patients with advanced IPF (DLCO < 35%) and otherwise operable non-small cell LC. Double platinum regimens and immunotherapy for metastatic disease were chosen by 25% and 32% of the respondents, respectively [162].

### Deconditioning and Sarcopenia

Deconditioning is a physical and/or psychological decline in function commonly experienced in all chronic diseases. Prolonged inactivity or reduced activity can affect nearly all systems of the body and can be regarded as an important comorbidity of chronic respiratory diseases such as COPD [163]. These changes are relevant, as they can affect the ability to do self-care, to walk, to engage in leisure activities and to work. Nishiyama et al.s’ prospective study showed that physical activity, expressed as steps taken, was significantly reduced in patients with IPF compared with healthy age-matched participants [164]. Lower daily physical activity also resulted in significantly worse survival in these patients. Others have reported similar findings in IPF [165] and other interstitial lung diseases [166]. Decreasing functional capacity is one of the main drivers of a poor quality of life (QoL) in ILD/IPF. It is primality influenced by exercise capacity, lung function and dyspnoea [167]. Other IPF comorbidities, such as CAD, COPD, PH, muscle mass, pain and depression, may also be deterministic [168]. Additional factors such as circulatory impairments and lower limb muscle dysfunction are also known to limit the exercise capacity of patients with ILD [169]. Deconditioning leads to loss of muscle mass (sarcopenia), which further leads to a vicious cycle of reduced activity [170].

Sarcopenia, defined as a progressive loss of muscle mass and strength, is associated with many adverse outcomes such as disability, poor QoL and death [171]. It can be regarded as a consequence of ageing processes, an important risk factor for IPF. Changes in body composition with skeletal muscle wasting, a major component of pulmonary cachexia, are associated with mortality [172] in COPD [173] and cancer [174]. Retrospective studies show that a low muscle mass in IPF may be a strong risk factor for all-cause mortality. In a study of lung transplant patients, the presence of ILD and COPD was predictive of muscle mass pre- and post-transplant [175]. Sarcopenia has been related to all-cause mortality, treatment comorbidities, QoL and reduced respiratory muscle strength; however, these associations are not well characterized in IPF.

The complex clinical syndrome of sarcopenia and deconditioning in IPF may clearly benefit from multimodal interventions, including a tailored therapeutic approach based on distinct wasting phenotypes. Early referral and participation in pulmonary rehabilitation programs may represent an important intervention to improve symptoms, physical activity and QoL in IPF [176]. Preliminary data suggest modest short-term benefits and recommend a longer-term maintenance programme to sustain these gains [177]. Behavioural interventions including social support and participation in support groups may reduce social isolation and help reinforce positive changes in IPF.

### Malnutrition and overnutrition

Various studies found that nutritional abnormalities, such as low body mass index (BMI), body weight loss, and vitamin D deficiency, seem to have negative prognostic significance in patients with IPF [178]. Several factors may have a negative impact on nutritional status in IPF patients, including an increased respiratory muscle load, the coexistence of hypoxemia, and physical inactivity leading to dynapenia (low muscle strength).

Despite the importance of nutritional abnormalities, their prevalence and impact in IPF is largely understudied.

According to the mean BMI values reported in multiple studies, in most studies, a high proportion of overweight/obese patients may be expected [179, 180]. In advanced stages of the disease, such conditions may become major issues because obesity is a contraindication to transplantation and is associated with an increased mortality risk in patients receiving bilateral lung transplantation [8].

The prevalence appears to be lower in underweight patients than in overweight patients; however, alterations in nutritional parameters suggestive of malnutrition were observed in nearly one-third of cases, particularly in advanced stages, in a recent study on French IPF patients [181].

The two currently available antifibrotic drugs nintedanib and pirfenidone can cause adverse events that may interfere with food intake and absorption, including diarrhoea, nausea and appetite loss [178].

Efforts should be made to reduce these adverse events to minimize the effect of chronic antifibrotic therapies on nutritional status.

## Is there a role for genetic testing in IPF?

Currently, the role of genetic testing in IPF is uncertain and limited to familial forms of pulmonary fibrosis, with a prevalence between 2 and 20% of all cases of IPF [182, 183]. Increasing evidence suggests that detecting hTERT/hTR pathogenic mutations may have clinical relevance, for instance, on the outcome of lung transplant recipients bearing these mutations [184]. It may also address the need for preventive measures in siblings of patients with pathogenic mutations, although there is no evidence that early diagnosis and preventive measures would change the course of the disease.

Furthermore, there is no consensus on the optimal workup with which to diagnose hTERT/hTR disease-associated variants. Relative simple assays such as flow fluorescence in situ hybridization (flow-FISH) or monochrome multiplex quantitative polymerase chain reaction (MM-qPCR) [185] can increase the suspicion of mutations in familial forms by assessing telomere length, but sequencing of the genes is still needed to confirm the diagnosis. Sequencing techniques, including whole-genome sequencing, are becoming more available in genetic labs, and their cost is becoming more affordable. Furthermore, whole-genome sequencing could potentially diagnose several gene mutations in the same patient, helping to shed light on the very complex interactions of genes involved in the pathogenesis of IPF [186]. Unfortunately, the clinical relevance and predictive value of these findings are unknown, and these tests cannot be recommended in the day-to-day clinical care of patients with IPF.

## Conclusions

IPF is a progressive fibrotic disease limited to the lungs; approved antifibrotic therapies may modify its relentless course by acting on a multitude of cellular pathways. However, IPF can also be viewed as a disease due to the interaction between ageing, genetic predisposition and environmental exposures such as cigarette smoke and is therefore often accompanied by comorbidities that affect the clinical phenotype and survival. Many comorbidities have substantial negative impacts on various outcome parameters in IPF. Therefore, the careful diagnosis and treatment of comorbidities may represent an excellent opportunity to optimize the survival of IPF patients. The broad range of comorbidities in IPF will require a holistic approach, contemplating all possible management options, including early identification and treatment.

It is possible that, in the future, elucidation of the fine genetic mechanisms underlying IPF and its comorbidities will enable better management of the disease, especially for familial forms.

## Data Availability

Not applicable.

## Abbreviations

IPF: Idiopathic pulmonary fibrosis
UIP: Usual interstitial pneumonia
HRCT: High-resolution computed tomography
hTERT: Human telomerase reverse transcriptase
hTR or TERC: Human telomerase RNA
NAF1: Nuclear assembly factor 1
DKC1: Dyskerin pseudouridine synthase 1
RTEL1: Regulator of telomere elongation helicase 1
ILD: Interstitial lung disease
CAD: Coronary artery disease
COPD: Chronic obstructive pulmonary disease
IL-17: Interleukin 17
HLA: Human leukocyte antigen
CTGF: Connective tissue growth factor
VTE: Venous thromboembolism
RCT: Randomized controlled trial
FVC: Forced vital capacity
DLCO: Carbon monoxide diffusion capacity
PDGF: Platelet-derived growth factor
RAGE: Receptor for advanced glycosylation end products
sRAGE: Soluble RAGE ligand
NKXS2-1: NK2 homeobox 1
CPFE: Combined pulmonary fibrosis and emphysema
PH: Pulmonary hypertension
mPAP: Mean pulmonary artery pressure
BNP: Brain natriuretic peptide
RHC: Right heart catheterization
6MWT: 6-Minute walking test
GERD: Gastro-oesophageal reflux disease
AE-IPF: Acute exacerbation of idiopathic pulmonary fibrosis
PPI: Proton-pump inhibitor
LARS: Laparoscopic anti-reflux surgery
OSA: Obstructive sleep apnoea
REM: Rapid eye movement
CPAP: Continuous positive airway pressure
LC: Lung cancer
SFTPA1: Surfactant protein A1
QoL: Quality of life
BMI: Body mass index
